# Detection of the Hematopoietic Stem and Progenitor Cell Marker CD133 during Angiogenesis in Three-Dimensional Collagen Gel Culture

**DOI:** 10.1155/2013/927403

**Published:** 2013-06-23

**Authors:** Masumi Akita, Kayoko Tanaka, Sachiko Matsumoto, Kumiko Komatsu, Keiko Fujita

**Affiliations:** ^1^Division of Morphological Science, Biomedical Research Center, Saitama Medical University, 38 Moroyama, Iruma-gun, Saitama 350-0495, Japan; ^2^Department of Anatomy, Saitama Medical University, 38 Moroyama, Iruma-gun, Saitama 350-0495, Japan

## Abstract

We detected the hematopoietic stem and progenitor cell marker CD133 using immunogold labeling during angiogenesis in a three-dimensional collagen gel culture. CD133-positive cells were present in capillary tubes newly formed from aortic explants in vitro. The CD133-positive cell population had the capacity to form capillary tubes. Lovastatin strongly inhibited cell migration from aortic explants and caused the degradation of the capillary tubes. The present study provides insight into the function of CD133 during angiogenesis as well as an explanation for the antiangiogenic effect of statins.

## 1. Introduction

CD133 was first isolated and cloned in 1997. CD133 expression was originally observed in hematopoietic stem and progenitor cells using a monoclonal antibody called AC133 [1] and neuroepithelial cells using a monoclonal antibody called prominin [2]. Gehling et al. [3] reported that the CD133-positive cell population consists of progenitor and stem cells that not only have hematopoietic potential but also have the capacity to differentiate into endothelial cells. Invernici et al. [4] reported that human fetal aorta contains vascular progenitor cells capable of inducing vasculogenesis and angiogenesis. Barcelos et al. [5] reported that human CD133 progenitor cells promote the healing of diabetic ischemic ulcers by paracrine stimulation of angiogenesis and activation of Wnt signaling. 

To grow, solid tumors require a blood supply. They recruit new blood vessels mainly by inducing the sprouting of endothelial cells from external vessels. Recent research in tumor biology shows that, in addition to recruiting vessels from outside the tumor, brain tumors produce endothelial cells for vessel formation within the tumor [6]. Wang et al. [7] reported that a glioblastoma cell population (CD144 and CD133 double positive) differentiated into endothelial cells and formed intracellular vacuolar structures in collagen gel. However, the biological function of CD133 in angiogenesis remains largely unknown.

For in vitro studies of angiogenesis, several culture techniques using matrix structures have been developed, including fibrin and collagen gels [8], Matrigel, collagen, fibrin, and plasma clots [9]. Collagen gel culture has been used widely and effectively for analyzing the biological process of angiogenesis [10–13]. Using a three-dimensional (3D) collagen gel culture, we have conducted electron microscopic studies [14, 15] and immunohistochemical studies of fibroblast growth factor- (FGF-) 2 and FGF-9 [16]. Additionally, we have used 3D collagen gel cultures to test angiogenic and anti-angiogenic agents (TNF-*α* and thalidomide) [17, 18] to study vascular injury after laser microdissection [19] and for profiling of DNA microarray gene expression during angiogenesis [20]. The 3D collagen gel culture system provides a simple and rapid method to analyze angiogenesis.

In the present study, we detected CD133 by immunogold labeling during angiogenesis in the 3D collagen gel culture system. Here, we show that CD133-positive cell population has a capacity to form capillary tubes.

## 2. Materials and Methods

### 2.1. Animals

ICR mice (male, 1 month old, *n* = 5; CLEA Japan, Inc.) and Wistar rats (male, 2 months old, *n* = 3; CLEA Japan, Inc.) were used for the experiment. The mice and rats were maintained according to the guidelines on the care and use of laboratory animals established by Saitama Medical University. These experiments were approved by the Animal Research Committee of Saitama Medical University.

### 2.2. Collagen Gel Culture of Mouse and Rat Aortae

The collagen culture technique used in the present study was modified from our previous technique [14, 15]. Thoracic aortae were obtained from mice and rats. Under a stereoscopic microscope, fibroadipose tissue and blood were removed from the aortae. The thoracic aortae were then serially cross-sectioned into ~2 mm rings. Four pieces were placed at the bottom of each tissue culture dish (35 mm; *n* = 25), overlaid with an even layer of reconstituted collagen solution (0.3% Cellmatrix type IA, Nitta Gelatin, Tokyo, Japan), and allowed to gel at 37°C for approximately 10 min. After the gels formed, they were overlaid with Ham's F-12 medium (Invitrogen Corp., Carlsbad, CA, USA), containing 20% fetal bovine serum (FBS), 1% nonessential amino acids, 100 units/mL penicillin, and 100 mg/mL streptomycin (Invitrogen Corp., Carlsbad, CA, USA), and cultured for 14 days in an incubator (95% air/5% CO_2_). The medium was replaced three times a week starting from day 3. Capillary tube formation was observed using a phase contrast microscope during the culture period. These experiments were performed three times.

### 2.3. Phase-Contrast Microscopy and Time-Lapse Imaging

Standard phase-contrast images were collected by using a phase-contrast inverted microscope (Nikon TE2000, Japan) and a CCD camera (ORCA-ER, Hamamatsu Photonics, Japan). For time-lapse experiments, the aortic rings were cultured as described above. Cells that grew out from the aortic rings were visualized using a phase-contrast inverted microscope equipped with a stage that was preheated to 37°C. The cells were maintained under 5% CO_2_ in a culture chamber during image acquisition, and images were recorded at 5 min intervals using an Aquacosmos imaging system (Hamamatsu Photonics, Japan).

### 2.4. Transmission Electron Microscopy

The cultured aortic rings were fixed in 0.1 M phosphate buffer (pH 7.2) containing 2.5% glutaraldehyde for 1 hour and then fixed in 0.1 M phosphate buffer (pH 7.2) containing 1% OsO_4_ for 1 hour. The rings were dehydrated in graded ethanol, embedded in epoxy resin, cut into ultrathin sections, and stained with uranyl acetate and lead citrate. The stained ultrathin sections were observed under a transmission electron microscope (JEM-1010, Tokyo, Japan). 

### 2.5. Rhodamine-Phalloidin and Lectin Histochemistry

After fixation in 4% paraformaldehyde/PBS, the cultured aortic rings were stained with rhodamine-phalloidin (Invitrogen Corp., Carlsbad, CA, USA) to determine the presence of F-actin, and FITC-conjugated endothelial-cell-specific tomato lectin (*Lycopersicon esculentum*, EY Labo, CA, USA), which selectively binds to fucose residues that are present on the endothelial cell surface, was used to label endothelial cells [21]. 

### 2.6. Immunohistochemical Detection of CD133

The cultured aortic rings were fixed in 4% paraformaldehyde/PBS. For the detection of CD133 on the cell surface, the rings were incubated overnight with CD133 antibody (rabbit polyclonal, Abcam, Tokyo, Japan) after treatment with 1% skim milk/PBS for 30 min, and then they were incubated with Alexa Fluor 488- and Nanogold (1.4 nm)-conjugated goat anti-rabbit IgG (Nanoprobes, Inc., Yaphank, NY) for 1 hour. The Nanogold signal was enhanced using GoldEnhance EM (Nanoprobes) at room temperature for 3–5 min for electron microscopy and 20–25 min for light microscopy. 

### 2.7. Effect of Lovastatin (Mevinolin) on Angiogenesis

Before and after tube formation, the effect of lovastatin (mevinolin from *Aspergillus* sp.) was tested. Lovastatin (mevinolin, M2147) supplied by Sigma has an empirical formula of C_24_H_36_O_5_ and is 2-methyl-1,2,3,7,8,8a-hexahydro-3,7-dimethyl-8-[2-(tetrahydro-4-hydroxy-6-oxo-2H-pyran-2-yl)-ethyl]-1-naphthalenyl ester butanoic acid. It is a white crystalline powder that is insoluble in water. A stock solution was prepared by dissolving it in 100% ethanol at a concentration of 12.3 mM [22]. Two different sets of experiments were designed as follows.

*Before Tube Formation*. Rat aortic rings were cultured in 35 mm dishes as described above. After 24 hours, aortic rings were cultured with 12.3 mM lovastatin or without lovastatin. Cultures were maintained at 37°C under 5% CO_2_ in a humidified incubator.

*After Tube Formation*. Rat aortic rings were cultured in 35 mm dishes as described above. After 10 days, lovastatin was added to the culture medium. Cultures were maintained at 37°C under 5% CO_2_ in a humidified incubator. 

### 2.8. Endothelial Cell Scraping

In a separate set of experiments, the trimmed thoracic aorta was cultured as follows. To visualise the intimal surface directly, the thoracic aorta was everted with a procedure that sequestered the adventitial cells and possible remnant microvessels of periaortic soft tissue inside the aortic tube [23]. The endothelial cells were scraped from the everted aorta with a sterile cotton swab. The everted aorta with/without endothelium was cut into small pieces and cultured in the same manner as previously described, and followed by 10% formalin fixation and Giemsa staining. 

## 3. Results

### 3.1. Capillary Tube Formation

#### 3.1.1. Phase-Contrast Microscopy

**Figure 1:**
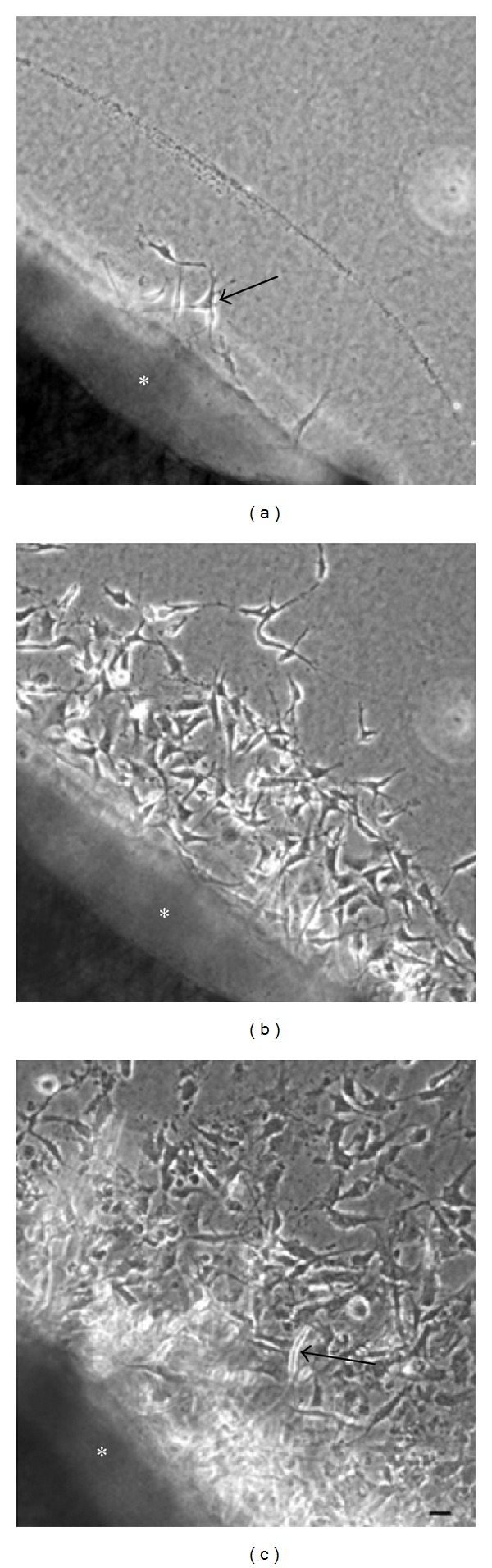
Phase-contrast microscopy. (a) After 2 days of cultivation, phase-contrast microscopy revealed fibroblastic cells (arrow) outgrown from a mouse aortic explant (∗) into a three-dimensional collagen gel. (b) After 5 days of cultivation, phase-contrast microscopy showed numerous fibroblastic cells outgrown from an aortic explant (∗). (c) After 7 days of cultivation, phase-contrast microscopy showed a tubular structure protruding (arrow) from an aortic explant (∗) into a three-dimensional collagen gel. (a), (b), and (c): Scale bar = 20 *μ*m.

 Microscope examination after as little as 2 days of culture revealed the presence of migrating cells proximal to the aortic ring in the collagen gel. These cells were spindle-shaped and their longitudinal axes were radially orientated toward the stump of the aortic ring (Figures 1(a) and 1(b)). After a 7-day culture period, capillary sprouts were recognizable (Figure 1(c)), although lumen formation was not observed in these early capillary structures. 

### 3.2. Time-Lapse Imaging of Capillary Tube Formation

**Figure 2:**
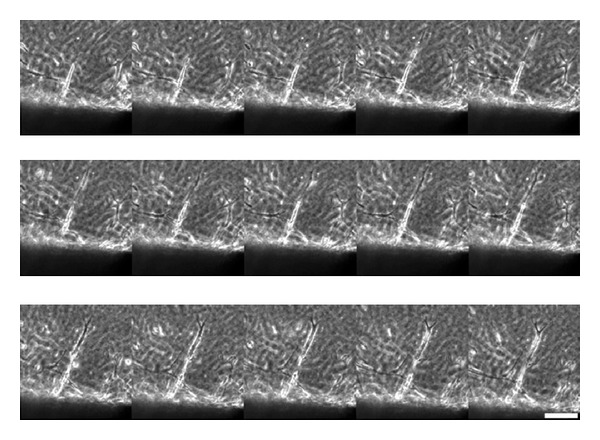
Time-lapse imaging of additional sprouts emerging and extending in a sequential manner from the leading edges of a newly formed capillary tube from the mouse aortic explant. Selected sequence from a time-lapse movie focusing on a single sprout. Note the protrusion of lamellipodia and continued migration of the leading cell. Scale bar = 50 *μ*m.

Time-lapse imaging was used to visualize the dynamic process of capillary tube formation from the aortic ring. Additional sprouts emerged and extended in a sequential manner from the leading edges of newly formed capillary tubes (Figure 2). 

### 3.3. Rhodamine-Phalloidin and Lectin Histochemistry

**Figure 3:**
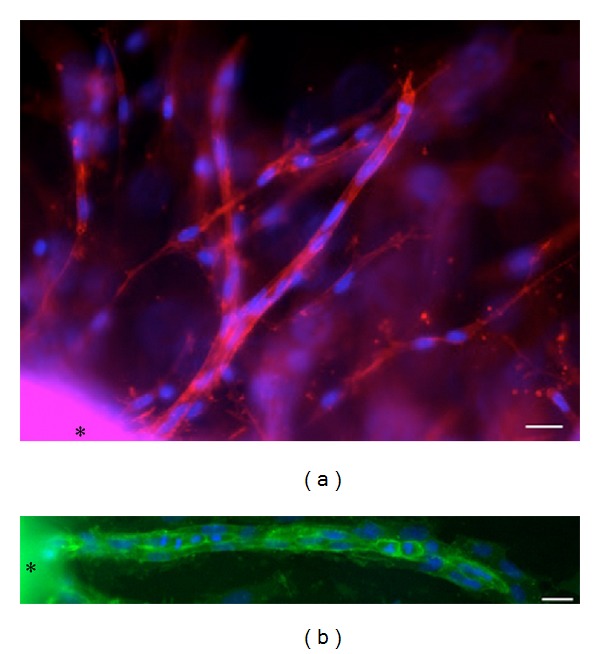
Rhodamine-phalloidin and lectin histochemistry. (a) After 11 days of cultivation, fluorescence microscopy showed capillary tubes that are stained with rhodamine-phalloidin for F-actin (purple-red) and 4′,6-diamidino-2-phenylindole (DAPI) nucleic acid stain (blue). The asterisk (∗) shows the mouse aortic explant. Scale bar = 20 *μ*m. (b) After 11 days of cultivation, the capillary tubes are strongly positive for FITC-conjugated endothelial-cell-specific tomato lectin staining (yellowish-green) and DAPI nucleic acid stain (blue). The asterisk (∗) shows the aortic explant. Scale bar = 20 *μ*m.

After 10 to 14 days in culture, elongated capillary tubes with branches were observed. Capillary tubes in the collagen gels were observed by rhodamine-phalloidin staining (Figure 3(a)). Capillary tubes that formed in the collagen gels were also strongly positive for tomato lectin (Figure 3(b)). 

### 3.4. Transmission Electron Microscopy

**Figure 4:**
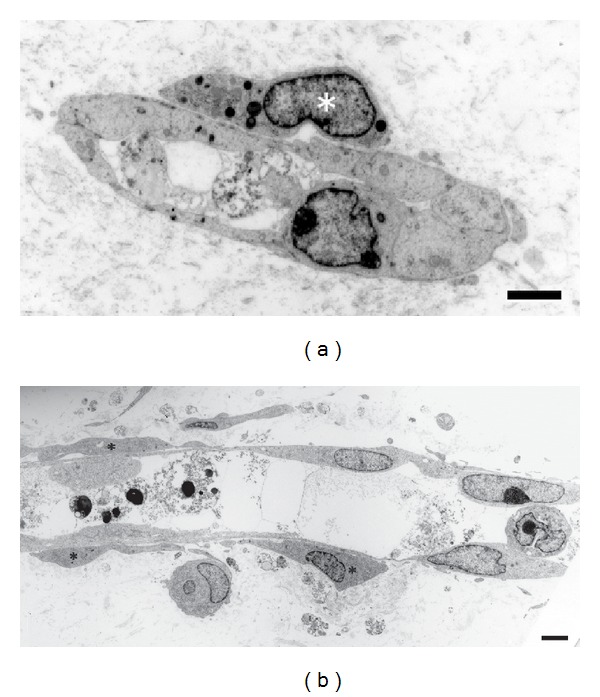
Transmission electron microscopy. (a) Electron microphotograph of a newly formed capillary tube from the mouse aortic explant in the collagen gel. A cross-section shows a capillary tube with a lumen that contains cell debris. Pericyte-like cells (∗) surround the tube. The edges of the cells are in contact with each other. Scale bar = 2 *μ*m. (b) Electron microphotograph of a longitudinal section of a capillary tube. Pericyte-like cells (∗) surround the tube. Scale bar = 2 *μ*m.

As demonstrated by cross-sectioning, endothelial cells of the capillary tubes formed tight contacts with each other, and pericyte-like cells were present on the outside of the endothelial cells (Figure 4(a)). The endothelial cells did not show any pores or gaps. Typical gap junctions and tight junctions were not observed. Cell organelles were present in large numbers, particularly in the thicker endothelial cells. Longitudinal sectioning revealed that endothelial cells and pericyte-like cells made close contact (Figure 4(b)).

### 3.5. Immunohistochemical Detection of CD133

**Figure 5:**
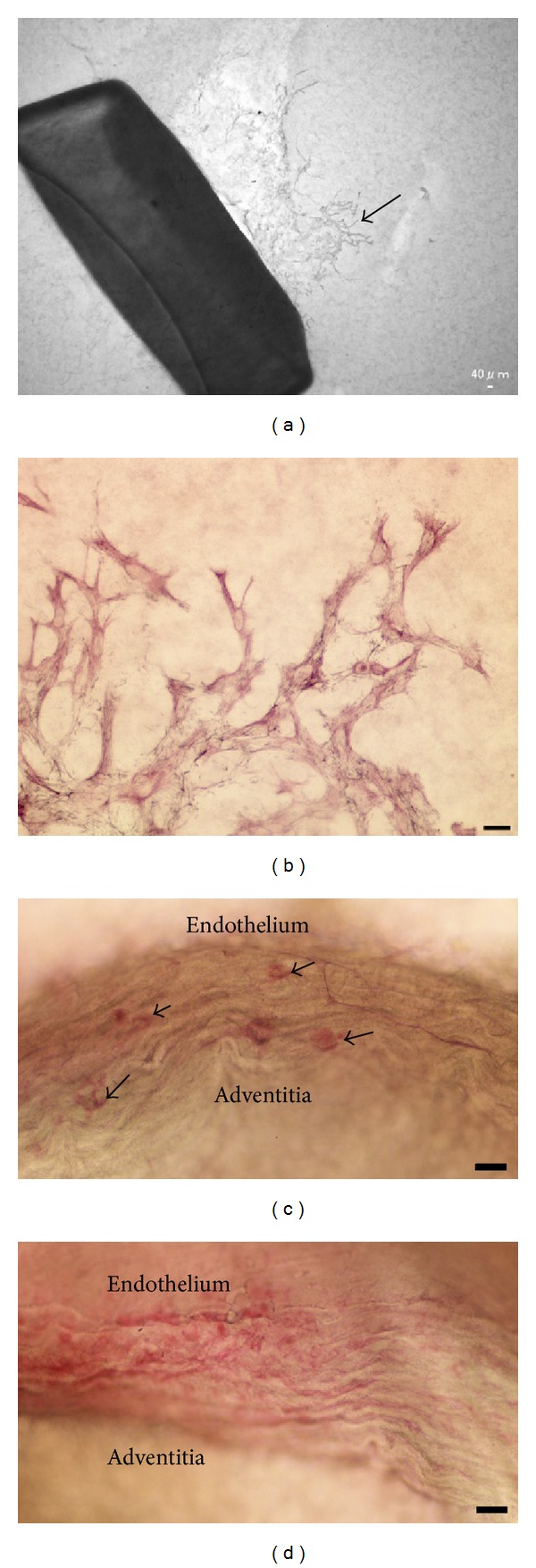
Immunohistochemistry of CD133. (a) In the early stages of culture (4-day culture), CD133-positive cells (arrow) were detected among cells migrating from the rat aortic explant. (b) Enlarged image of migrating CD133-positive cells. Scale bar = 20 *μ*m. (c) CD133 expression (arrows) was found in the smooth muscle layer and near the adventitia. Scale bar = 20 *μ*m. (d) CD133 expression was also found in the endothelium and the smooth muscle layer. Scale bar = 20 *μ*m.

**Figure 6:**
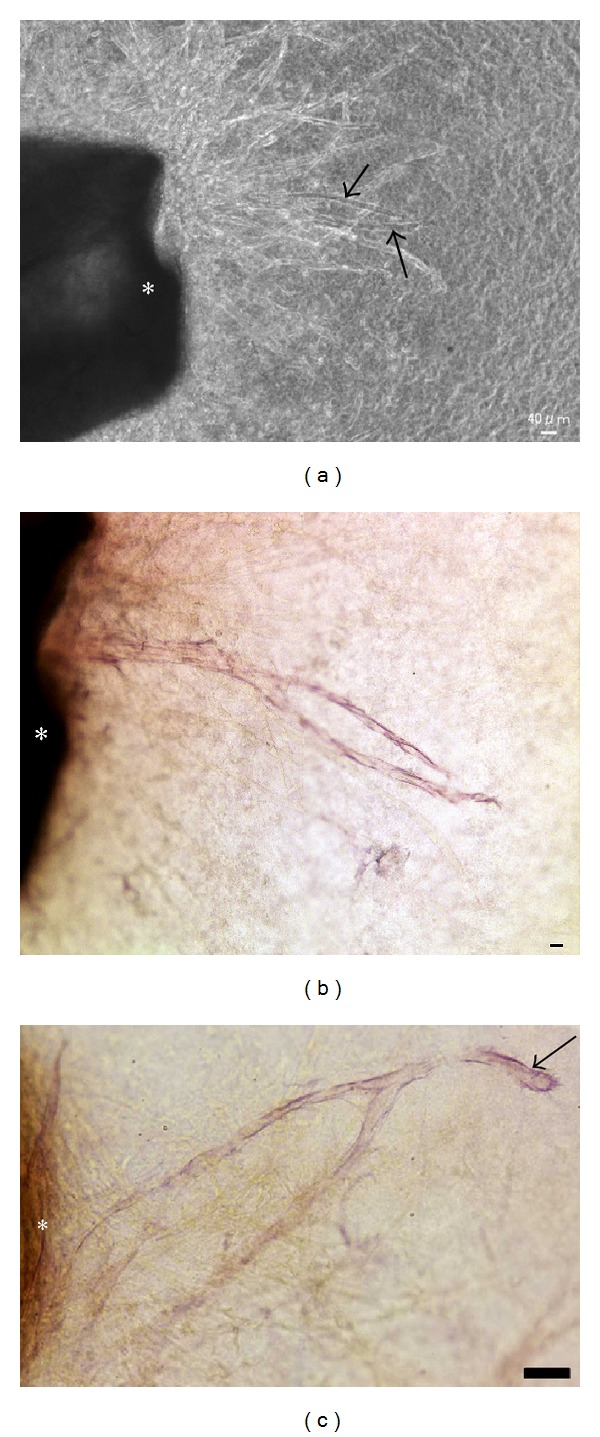
CD133 immunoreactivity in capillary tubes. (a) In the later stages of culture (9-day culture), capillary tubes formed. Phase-contrast microscopy showed that many tubes were formed from the rat aortic explant. Arrows indicate CD133-positive tubes among CD133-negative tubes. Rat aortic explant (∗). (b) CD133-positive cells were present in a tubular pattern. Photograph showing CD133-positive tubes indicated by arrows in (a). Rat aortic explant (∗). Scale bar = 20 *μ*m. (c) CD133 expression was clearly found in the tip region (arrow) of the tube rather than the stalk region near the rat aortic explant (∗). Scale bar = 20 *μ*m.

**Figure 7:**
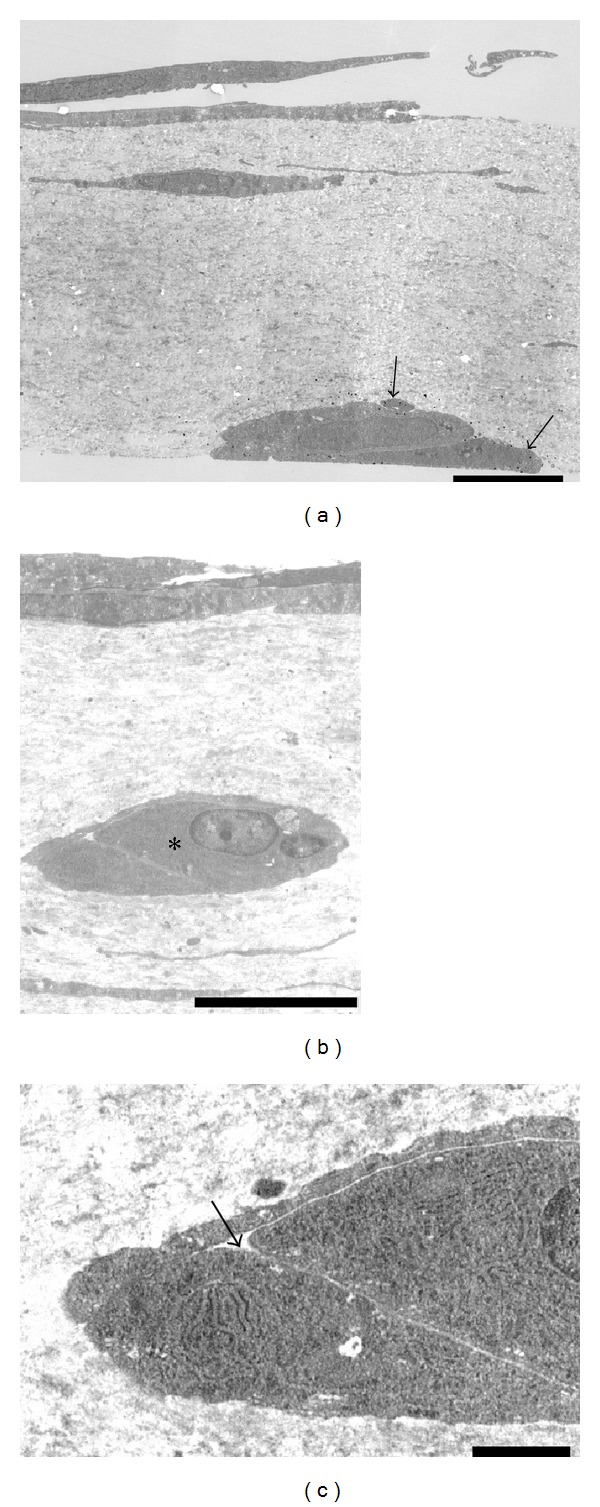
Immunoelectron microscopy of CD133. (a) At the bottom side of the collagen gels, CD133-positive cells made contact with each other (arrows). Cell organelles were sparse in the cells. Scale bar = 5 *μ*m. (b) CD133-negative cells made contact with each other in the middle layer of the collagen gels (∗). Scale bar = 5 *μ*m. (c) Enlargement of (b) (∗). These cells formed intercellular vacuolar structures (arrow). Cell organelles, such as the rough endoplasmic reticulum, were rich in these cells. Scale bar = 1 *μ*m.

In the early stages of culture, CD133-positive cells were detected among cells migrating from the aortic ring (Figures 5(a) and 5(b)). CD133 expression was also found within the aortic wall (Figures 5(c) and 5(d)). In the later stages of culture, capillary tubes formed. CD133-positive cells were present in a tube-like pattern (Figures 6(a) and 6(b)), CD133 expression was found in both the tip and stalk regions. The leading edge of the capillary tube was strongly positive for CD133 (Figure 6(c)). Electron microscopic observation revealed CD133 expression in cells located on the bottom of the collagen gels (Figure 7). 

### 3.6. Effect of Lovastatin (Mevinolin) on Angiogenesis

**Figure 8:**
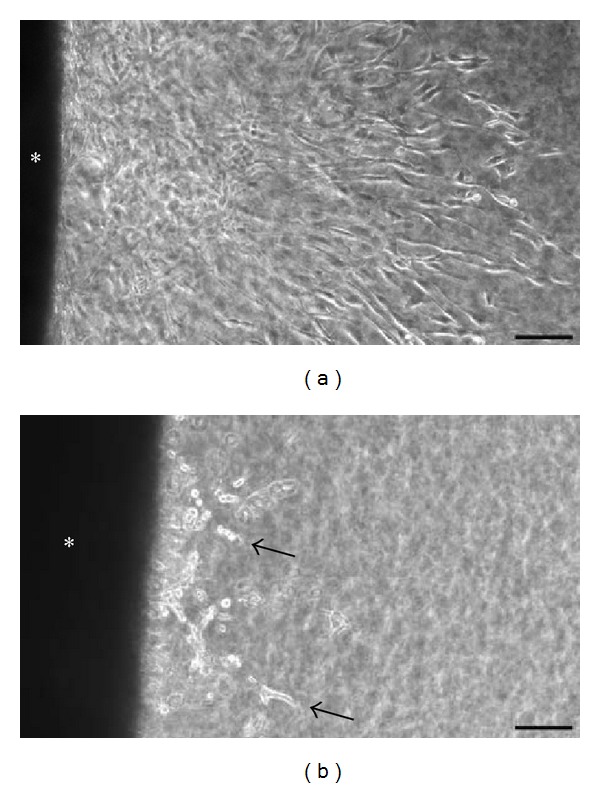
Effect of lovastatin (mevinolin) before tube formation. (a)-(b) When rat aortic rings were cultured with lovastatin, cell migration (arrows) was strongly inhibited relative to the control. (a) = control, (b) = lovastatin, aortic explant (∗). Scale bar = 100 *μ*m.

*Before Tube Formation*. When aortic rings were cultured with lovastatin, cell migration was strongly inhibited relative to the control (Figures 8(a) and 8(b)).

**Figure 9:**
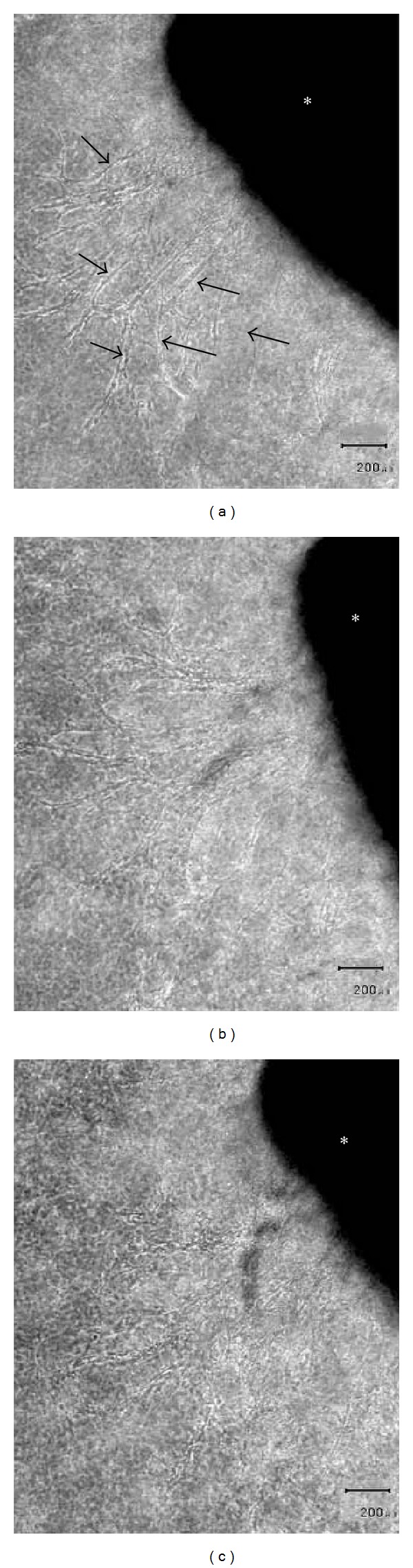
Effect of lovastatin (mevinolin) after tube formation. (a)–(c) After tube formation, lovastatin treatment induced the degradation of newly formed capillary tubes (arrows). (a) Before Lovastatin treatment; (b) after 24 hours; (c) after 48 hours; rat aortic explant (∗). Scale bar = 200 *μ*m.

**Figure 10:**
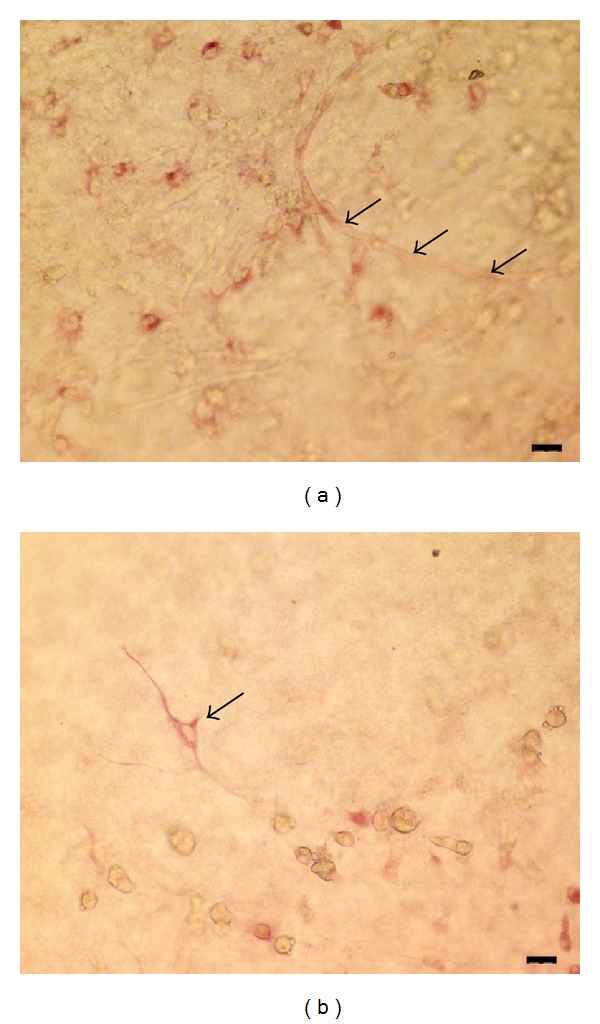
(a) Cell-cell adhesion was diminished, and many CD133-positive cells adopted a round morphology. Arrows indicated the degradation of capillary tubes. Scale bar = 20 *μ*m. (b) Some polygonal cells with cell processes maintained their morphology (arrow). Scale bar = 20 *μ*m.

*After Tube Formation*. Lovastatin treatment induced the degradation of newly formed capillary tubes (Figures 9(a), 9(b), and 9(c)). Cell-cell adhesion was diminished, and the morphology of many CD133-positive cells changed to an oval shape (Figure 10(a)), although some polygonal cells with cell processes maintained their morphology (Figure 10(b)).

### 3.7. Effect of Endothelial Cell Scraping

**Figure 11:**
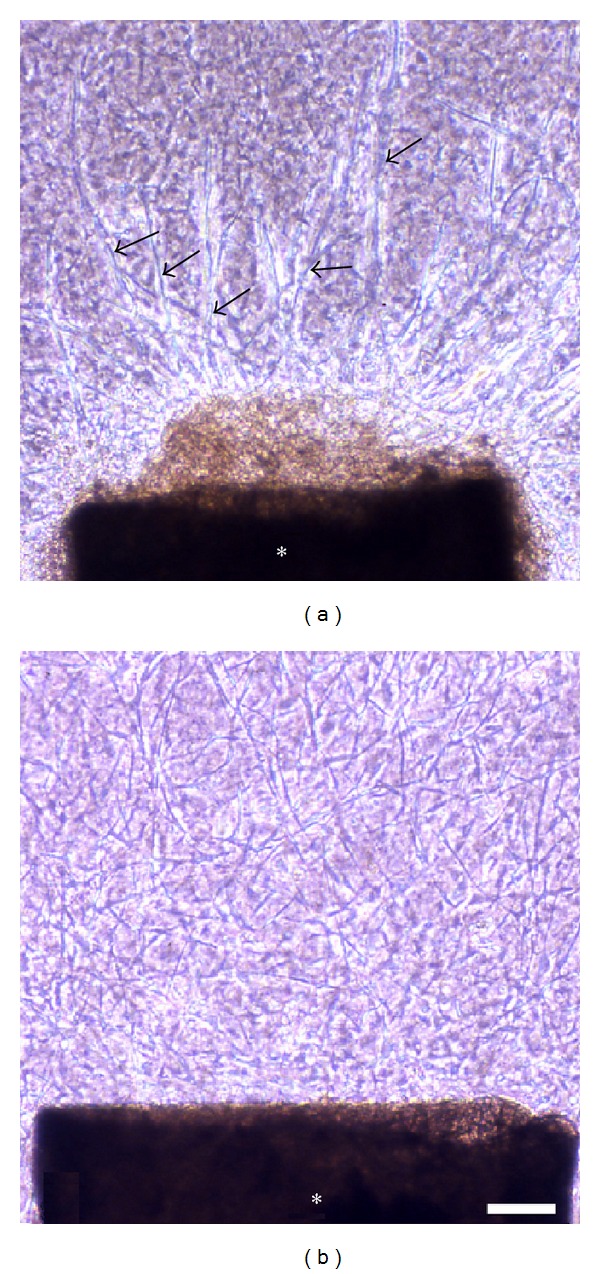
(a) A control culture from the rat aorta explant. Many capillary tubes (arrows) are present. (b) A deendothelialized rat aortic explant that has spindle-shaped cells migrating into collagen gels. However, capillary tube formation did not occur. Rat aortic explant (∗), Giemsa stain. Scale bar = 200 *μ*m.

For the everted aorta with intact epithelium, spindle-shaped cells migrated into the collagen gels, and capillary tube formation occurred in a similar manner (Figure 11(a)). After endothelial cell scraping, spindle-shaped cells migrated into the collagen gels from the everted aorta, even without the presence of epithelial cells. However, capillary tube formation did not occur (Figure 11(b)).

## 4. Discussion

Recently, LS-7 (amino acid sequence: LQNAPRS), which is a specific binding peptide that targets mouse CD133, was screened and identified for the first time using phage-displayed peptide library technology [24]. However, the biological function of CD133 remains unclear. CD133 expression is not restricted to the neuroepithelial and hematopoietic stem and progenitor cells in which it was originally observed; it also extends to several epithelial and nonepithelial cell types. CD133 is also widely used as a marker for cancer stem cells (CSCs) in many different types of solid tumors including colon [25, 26], brain [27, 28], skin [29], pancreatic [30], liver [31–33], and prostate [34] tumors. Wang et al. [7] and Ricci-Vitiani et al. [35] presented evidence that tumor-derived endothelial cells arise from tumor stem-like cells. Wang et al. [7] found that a glioblastoma cell population that could differentiate into endothelial cells and form intracellular vacuolar structures in collagen gels was enriched in cells expressing CD133. Although the possibility of endothelial differentiation of tumor cells has been suggested in lymphoma, myeloma, chronic myeloid leukemia, breast cancer, and neuroblastoma [36–40], the angiogenic activity of CSCs has not been investigated in other types of tumors. Because glioblastoma is one of the most vascular-rich tumors, further investigation is needed to evaluate the differentiation of CSCs into endothelial cells. 

In the present study, we demonstrated that CD133-positive cells were present in the newly formed capillary tubes. Wang et al. [7] suggested that the differentiation of CSCs into endothelial cells may be mediated by signaling pathways involving two proteins: vascular endothelial growth factor (VEGF) and notch. Wang et al. [7] proposed that notch regulates the initial differentiation of cancer stem cells to endothelial progenitor cells, whereas VEGF selectively affects the differentiation of endothelial progenitors to tumor-derived endothelial cells. We showed that the leading edge of the capillary tube was strongly positive for CD133, which suggests that CD133-positive cells are involved in the elongation and/or branching of capillary tubes. Mature endothelial cells do not express CD133. CD133 is expressed by endothelial precursors and rapidly lost upon differentiation into mature endothelial cells [41]. It is therefore likely that the newly formed capillary tubes that consist of CD133-positive cells are immature. Soda et al. [42] reported that hypoxia-inducible factor-1 (HIF-1) is an important enhancer of EC differentiation of tumor cells and that the formation of tumor-derived endothelial cells is independent of VEGF. Hypoxic conditions may have thus resulted in the formation of CD133-positive capillary tubes at the bottom of the collagen gels.

The present study showed that CD133-positive cells were also present within aortic explants. Zengin et al. [43] reported the existence of endothelial precursor and stem cells in a distinct zone of the vascular wall that are capable to differentiate into mature endothelial cells, hematopoietic cells, and local immune cells such as macrophages. This zone has been identified to be loculated between the smooth muscle and the adventitial layer of the adult human vascular wall. Progenitor cells isolated from the adventitia of both murine and human blood vessels have the potential to form endothelial cells, mural cells, osteogenic cells, and adipocytes. These progenitors appear to cluster at or near the border zone between the outer media and inner adventitia [44]. After scraping the epithelium, spindle-shaped cells migrated into the collagen gels from both ends of the everted aorta. However, capillary tube formation did not occur. In this aortic culture, it is likely that the primary source of newly formed capillary tubes is the intimal endothelial cells. Nicosia [12] also noted that rat carotid artery explants failed to generate an angiogenic response when completely deendothelialized with a balloon catheter, whereas control arteries with an intact intimal endothelium produced microvessels from their resected ends. At the present time, it is unclear whether the primary source of newly formed capillary tubes is derived from the distinct zone between the smooth muscle and the adventitial layer or from the intimal endothelial cell layer. Further studies are needed to clarify these issues.

The present study also showed that lovastatin strongly inhibited cell migration from the aortic explant. The mechanism of inhibition of cell migration is considered as follows. Lovastatin is a potent inhibitor of hydroxymethylglutaryl-coenzyme A (HMG-CoA) reductase. The inhibition of HMG-CoA reductase, which is involved in lipid metabolism, causes the lipids necessary for the normal membrane functioning to become defective, and further impairment is seen in their adhesive properties. The adhesion of lipids is mediated through integrins, which are necessary for cell motility and migration through the extracellular matrix in the process of invasion [22]. Clinical data indicate that statin-treated patients have diminished intraplaque angiogenesis [45], which suggests that statins have angiostatic effects in vivo. It is also intriguing that statins have been reported to reduce the growth and spread of many cancers [46, 47], which may be related to inhibition of angiogenesis [48]. The present study also showed that lovastatin cased the abrogation of cell-cell adhesion and degradation of capillary tubes. Khaidakov et al. [48] suggested that statins through VE-cadherin stimulation modulate cell-cell adhesion and diminish the ability of cells to proliferate and migrate. Recently, Koyama-Nasu et al. [49] reported that CD133 interacts with plakoglobin (also known as c-catenin), a desmosomal linker protein. They further demonstrate that knockdown of CD133 by RNA interference (RNAi) results in the downregulation of desmoglein-2, a desmosomal cadherin, and abrogates cell-cell adhesion and tumorigenicity of clear cell carcinoma of the ovary stem cells. In addition, we reported that the cholesterol chelating agent, methyl-*β*-cyclodextrin, diminished cell adhesion by decreasing desmosomes and intercellular digitations. A decrease in the cholesterol level may perturb CD133 membrane localization [50]. Modulation of cell-cell adhesion may help to explain the degradation of the capillary tubes. The present study thus provides insight into the function of CD133 during angiogenesis and provides an explanation for the anti-angiogenic effect of statins.

## 5. Conclusions

The CD133-positive cell population has the capacity to form capillary tubes. The present study provides a useful method for determining the function of CD133 during angiogenesis.
